# Genomic variation in cline shape across a hybrid zone

**DOI:** 10.1002/ece3.375

**Published:** 2012-10-01

**Authors:** Sarah E Kingston, Robert W Jernigan, William F Fagan, David Braun, Michael J Braun

**Affiliations:** 1 Program in Behavior, Ecology, Evolution, and Systematics, University of MarylandCollege Park, Maryland, 20742, USA; 2 Vertebrate Zoology, Smithsonian Institution, National Museum of Natural HistoryWashington, District of Columbia, 20013, USA; 3 Department of Mathematics and Statistics, American UniversityWashington, District of Columbia, 20016, USA; 4 Department of Biology, University of MarylandCollege Park, Maryland, 20742, USA; 5 Plateau Land and Wildlife ManagementDripping Springs, Texas, 78620, USA

**Keywords:** AFLP, gene flow, hybridization, introgression, isozyme, mitochondrial DNA, *Pipilo maculatus*, *Pipilo ocai*, tension zone model, towhee

## Abstract

Hybrid zones are unique biological interfaces that reveal both population level and species level evolutionary processes. A genome-scale approach to assess gene flow across hybrid zones is vital, and now possible. In Mexican towhees (genus *Pipilo*), several morphological hybrid gradients exist. We completed a genome survey across one such gradient (9 populations, 140 birds) using mitochondrial DNA, 28 isozyme, and 377 AFLP markers. To assess variation in introgression among loci, cline parameters (i.e., width, center) for the 61 clinally varying loci were estimated and compiled into genomic distributions for tests against three empirical models spanning the range of observed cline shape. No single model accounts for observed variation in cline shape among loci. Numerous backcross individuals near the gradient center confirm a hybrid origin for these populations, contrary to a previous hypothesis based on social mimicry and character displacement. In addition, the observed variation does not bin into well-defined categories of locus types (e.g., neutral vs. highly selected). Our multi-locus analysis reveals cross-genomic variation in selective constraints on gene flow and locus-specific flexibility in the permeability of the interspecies membrane.

## Introduction

The phenomenon of divergence in the face of gene flow and the role of hybridization in the evolutionary process are of collective interest (Barton 2001; Doebeli and Dieckmann 2003; Grahame et al. 2006; Strasburg and Rieseberg 2008). Hybridization between closely related species can inform our understanding of evolutionary processes on population through phylogenetic levels. Steep genetic clines across stable areas of hybridization are the classic examples of the “tension zone” model of hybrid zones, where the transition between parental forms is maintained by a balance between endogenous selection and dispersal, arbitrated by linkage disequilibrium (Barton and Hewitt 1985, 1989). In this framework, hybrids will, on average, be less fit than the parental types. Such fitness differences arise, in part, because the new recombinant genotypes of hybrid individuals have never been tested on the opposite genotypic backgrounds even though parental lineages have been subject to generations of selective pressure. “Speciation genes” can deepen this valley of hybrid fitness through effects on reproductive success (Wu and Ting 2004). On the other hand, some recombinant genotypes may be advantageous in alternative environments, in which case even strong selection against early generation hybrids will not prevent the movement of these alleles across many hybrid zones (Moore 1977; Barton and Bengtsson 1986; Pialek and Barton 1997; Barton 2001; Strasburg and Rieseberg 2008). If hybridization is frequent and ongoing, neutral alleles may also leak across a hybrid zone; however, sufficiently strong linkage disequilibrium can oppose such allelic escape (Gavrilets 1997). Thus, the relative freedom of any single allele to introgress is related to the strength of selection on, and linkage disequilibrium to, favored or disfavored alleles.

Research focusing on divergent parental species and diagnostic markers accentuating these differences may underestimate levels of interspecies introgression (Brumfield et al. 2001). The classic assertion that avian hybrid zones, in particular, “swallow” the interspecies genetic mixing via strong selection against hybrid offspring may arise because many of the well-studied hybrid zones are between highly differentiated species exhibiting steep clinal variation (Moore 1977). Although the conscious choice of diagnostic markers facilitates identification of parental types and hybrids, it may also perpetuate a focus on markers and traits under strong purifying selection (Sattler and Braun 2000; Brumfield et al. 2001). This level of selection may not be representative of the rest of the genome, which may experience greater gene flow. To provide a more comprehensive portrait of introgression across hybrid zones, a dense genome survey is required (Rieseberg et al. 2003). Such a survey, which allows inference of cline shape parameters from spatial changes in allele frequency across hybrid transects for many loci, can provide estimates of the degree to which introgression varies among loci across the genome.

Divergence despite gene flow between incipient or young species may be an important part of the speciation process (Hey 2006). Empirical evidence of introgression due to hybridization is growing. Allelic introgression between species has been demonstrated in several systems, including *Drosophila,* butterflies, mice, and reef fish (Wang et al. 1997; Crow et al. 2007; Mallet et al. 2007; Teeter et al. 2008; Gompert et al. 2010a,b). Among birds, mitochondrial and nuclear markers suggest that cryptic introgression between golden-winged and blue-winged warblers is common (Vallender et al. 2007). Introgression of plumage and microsatellite markers has occurred across a Panamanian manakin hybrid zone (Brumfield et al. 2001; Yuri et al. 2009). Several avian hybrid zones are moving spatially where one species is encroaching upon the other through hybridization and introgression. These include black-capped and Carolina chickadees, Townsend's and hermit warblers, lazuli and indigo buntings, and blue-winged and golden-winged warblers (Sattler and Braun 2000; Rohwer et al. 2001; Owen-Ashley and Butler 2004; Bronson et al. 2005; Vallender et al. 2007; Carling and Zuckerberg 2011).

In contrast with the moving hybrid zones, stable regions of hybridization between two species of towhee, *Pipilo maculatus* (spotted towhee) and *P. ocai* (collared towhee), in Mexico have been intensively delineated using morphological characters (Sibley 1950, 1954; Sibley and West 1958; Sibley and Sibley 1964; Braun 1983) and isozyme loci (Braun 1983). Characterization of specimens dating back to the 19th century affirms that morphological intermediacy within these towhee populations has been geographically stable over a period of at least 150 years (Sibley 1950; Braun 1983).

**Figure 1:**
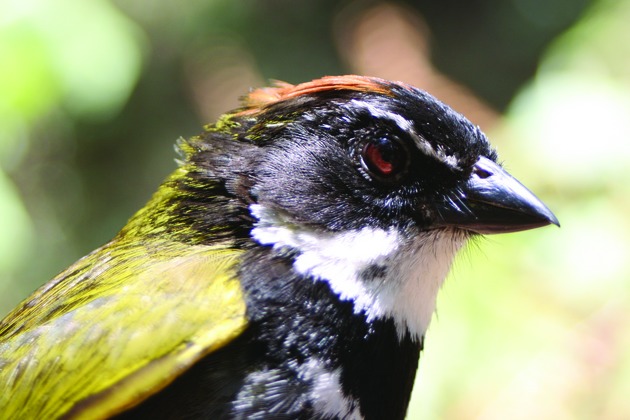
Photograph of *Pipilo ocai*, Nevado de Colima, Jalisco, Mexico (photograph by Sarah Kingston).

*P. maculatus* and *P. ocai* are strikingly different in plumage and have substantial mitochondrial DNA (mtDNA) sequence divergence (∼5.4% in cytochrome *b*, ND 2, and the control region) (Zink et al. 1998). In general, *P. ocai* prefer higher elevation (∼3000–3700 m), cooler, moister, coniferous (fir/pine forest) habitat, whereas *P. maculatus* prefer a lower elevation (∼2100–3300 m), warmer, drier, more open brushy pine/oak forest mixture. Sibley describes several major axes of hybridization: (1) the Teziutlán gradient running north – south down the Sierra Madre Oriental, (2) the Transplateau gradient running west – east across the transvolcanic belt of central Mexico, and (3) a smaller gradient running north – south along the southern edge of the Sierra Madre Occidental (Sibley 1950). These areas of hybridization are not simply intrusions of parental types into shared habitat, but rather extensive hybrid swarms where each population in the gradient demonstrates intermediate plumage, song, and behavior (Sibley 1950, 1954; Sibley and Sibley 1964; Braun 1983). Remarkably, in addition to the three hybrid axes, there are sympatric populations of *P. ocai* and *P. maculatus* on Mt. Orizaba and south into Oaxaca, which bear little morphological evidence of hybridization, just tens of kilometers away from two of the gradients (Sibley 1950, 1954) (exemplary photo of *P. ocai* parental type, Fig. 1).

The towhee species may have diverged in allopatry due to different Pleistocene glacial refugia, and come back into contact sometime in the last 10,000 years. The reestablished contact may predate the most recent glacial retreat and the two lineages may have been in and out of contact in a cyclical fashion. Although anthropogenic impact on forest habitat may have contributed to reestablishing contact between the two species, the area of species contact has been under anthropogenic influence for at least 3000–5000 years (Sibley and Sibley 1964). Thus, the contact and subsequent hybridization between *P. maculatus* and *P. ocai* is likely at least 3000 years old, and probably older. Given the several stable areas of hybridization, why hasn't gene flow swamped the species differences? Is selection acting in a classic tension zone manner to constrain the interspecies exchange of genes, or are some alleles free to flow between species despite the retention of other genetic differences? The towhee hybrid contact offers a great system in which to address such evolutionary questions, as these hybrid gradients are often proffered as a classic case of the breakdown of reproductive isolation (Mayr 1963).

Given the presence of sympatric towhee populations near the hybrid gradients, it has also been theorized that the morphological variation demonstrated among these towhee populations is not related to hybridization at all. The “character displacement/social mimicry hypothesis” posits plumage character displacement causing enhanced differences among sympatric populations of *P. maculatus* and *P. ocai,* whereas allopatric populations are free from such selective divergence and the plumage differences are weakened or lost entirely (Brown and Wilson 1956). In these allopatric populations, social mimicry is hypothesized between towhees and the *ocai*-like chestnut-capped brush finch (*Arremon brunneinucha)*, causing convergence of plumage patterns between either *Pipilo* species and *A. brunneinucha* in order to increase interspecies territoriality and reduce competition for resources between these ecologically similar birds (Cody and Brown 1970). Thus, this hypothesis predicts that *P. maculatus* populations in allopatry with *P. ocai* are not only free to drift to a more *ocai*-like appearance due to release from character displacement, but are driven to a more *ocai*-like appearance by territorial niche-partitioning with *A. brunneinucha,* consequently resembling hybrids between *maculatus* and *ocai*. In this case, rather than mixed genetic signatures in a gradient across the morphological areas of apparent hybridization, we would expect to see *P. maculatus*-like genotypes even as the appearance of towhee populations shift toward *ocai*-like.

Herein, we assess genome-wide variation in permeability across the interspecies genetic membrane. We will test the contrasting predictions expected under the interspecies hybridization and character displacement/social mimicry hypotheses. We also aim to assess the genomic distribution of cline parameters across the hybrid interface and the variation in signature of the effects of selection and introgression. We hypothesize there is not one universal genome-wide clinal model, but diversity in cline extent and shape demonstrating substantial variance in permeability of the species boundary. To evaluate the heterogeneity of underlying factors influencing the system, we will test the extent and strength of a cohesive cross-locus signal by attempting to pinpoint natural groupings among loci.

## Methods

### Sampling

**Figure 2:**
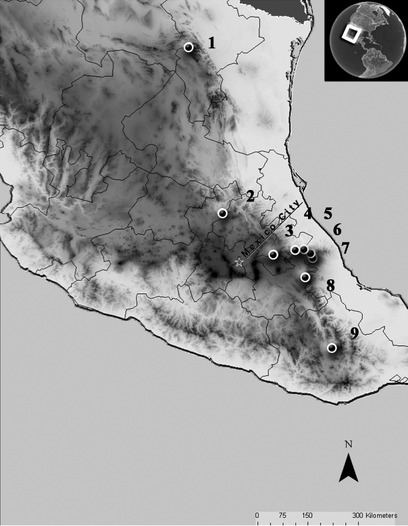
Sampling locations of towhee populations along the Teziutlán hybrid gradient in the Sierra Madre Oriental. Numbered locations are detailed in Table 1.

Nine populations (140 individual birds, 15.56 mean sampled per population) were sampled across the 1156 km Teziutlán transect through a ribbon of Sierra Madre Oriental montane forest (Fig. 2, Table 1). Collection sites ranged from 1956 to 3781 m in elevation, exhibiting pine/oak scrub habitat at lower elevations and pine/fir habitat at higher elevations. Birds were collected using mist nets or shotgun, tissue was collected for cryopreservation, and vouchered museum skin specimens were prepared (Braun 1983). The morphological variation in these samples encompasses the full range from pure *P. maculatus* to pure *P. ocai* (Braun 1983) based on Sibley's six character hybrid index (Sibley 1950, 1954) (Table 1). Tissues were deposited at the US National Museum of Natural History and vouchers were deposited at Louisiana State University Museum of Natural Science (Table SA1).

**Table 1:** Local area, plumage type, mean population hybrid index (plumage), coordinates, location elevation, and sample size of sampling locations ordered from north to south through the Teziutlán hybrid gradient

Number	Location	Plumage type	Hybrid index	Lat	Long	Elevation (m)	Km from no. 1	Sample *N*
1	Coahuila	*Pipilo maculatus*	24.0	25.250	−100.450	2902	0	16
2	Queretaro	*Pipilo maculatus*	22.8	20.783	−99.567	3063	538	17
3	Tlaxco	hybrid	21.5	19.667	−98.167	2667	728	17
4	E. Huichautla	hybrid	17.8	19.783	−97.600	2355	793	14
5	Teziutlán	hybrid	14.9	19.817	−97.367	1956	818	15
6	R. Palenquillo	hybrid	4.4	19.650	−97.117	2383	846	16
7	Cofre de Perote	hybrid	3.4	19.567	−97.100	3028	856	15
8	Orizaba	*Pipilo ocai*	0.2	19.050	−97.308	3781	917	16
9	Oaxaca	*Pipilo ocai*	0.0	17.167	−96.633	2760	1156	14
	Total							140

### Molecular analyses

DNA was extracted from tissue using a standard proteinase K/phenol-chloroform protocol similar to that of Sattler and Braun (2000). DNA concentration was quantified using a Nanodrop ND-1000 spectrophotometer, and chain length was assessed using agarose gel electrophoresis.

### Mitochondrial DNA

Mitochondrial DNA haplotypes were scored from Southern blots (Southern 1975) as described by Sattler and Braun (2000). Briefly, total genomic DNA was digested using restriction enzymes and the fragments separated by size in agarose gels. The digests were transferred to nylon membranes and mtDNA fragments for each individual visualized using hybridization with radiolabelled mtDNA from House Finch (*Carpodacus mexicanus*). An initial survey of 31 restriction enzymes revealed that 12 enzymes showed diagnostic differences between parental populations of *P. ocai* and *P. maculatus*. Three diagnostic enzymes (*Bcl I*, *Bgl I*, and *Hae III*) were then used to score all individuals.

### Isozymes

Protein electrophoresis was completed on homogenized muscle, heart, and liver tissue according to Braun (1983) yielding 39 isozyme loci. When loci exhibited more than two alleles, clinal patterns were visualized using the allele demonstrating the greatest variation in frequency and/or the greatest frequency in population 1.

### AFLP

The AFLP protocol was run according to Vos et al. (1995) with modifications according to Kingston and Rosel (2004). The restriction enzymes used were *Taq*I and *Eco*RI. Digestions were loaded with 200 ng of genomic DNA (50 uL total digestion reaction volume). Ten selective primer combinations were utilized (Table 2). Selective *Eco*RI primers were labeled with fluorescent dye and fragment sizes were assessed on an ABI 3100 genetic analyzer via capillary electrophoresis with an internal size standard. Electropherograms were visualized and fragment presence/absence scored in ABI GeneMapper 4.1 software. To avoid artifacts, the Kingston and Rosel (2004) scoring protocol was utilized.

**Table 2:** Adapters and primers used in the AFLP assay

Oligo name	Sequence
EcoRIadp_F	CTCGTAGACTGCGTACC
EcoRIadp_R	AATTGGTACGCAGTCTAC
TaqIadp_F	GACGATGAGTCCTGAC
TaqIadp_R	CGGTCAGGACTCAT
EcoRIpresel	GACTGCGTACCAATTCA
TaqIpresel	GATGAGTCCTGACCGAA
EcoRI + AAC	GACTGCGTACCAATTCAAC
EcoRI + AAG	GACTGCGTACCAATTCAAG
TaqI + AAC	GATGAGTCCTGACCGAAAC
TaqI + AAG	GATGAGTCCTGACCGAAAG
TaqI + ACA	GATGAGTCCTGACCGAACA
TaqI + ACT	GATGAGTCCTGACCGAACT
TaqI + AGA	GATGAGTCCTGACCGAAGA

### Analyses of cline position and shape

Population allele frequencies of AFLP markers were inferred from the dominant binary data using the assumptions of Hardy–Weinberg equilibrium (HWE; frequency of the null state, 0, represents q^2^). A simulation using the co-dominant isozyme data was completed to assess the least-biased allele frequency estimation method for dominant markers in the towhee system. Simulated dominant markers were re-sampled based on the empirical population allele frequencies of the 28 polymorphic isozyme loci. Allele frequencies were estimated using the HWE square root method and Bayesian inference through the program Hickory (Holsinger and Lewis 2003). These allele frequencies were then compared with the co-dominant data from which the dominant scores were derived. HWE square root method provided the least-biased estimates (mean [± SD] difference between co-dominant allele frequency and frequency estimated from dominant scores was 0.015 [± 0.032]). In order to measure differentiation between parental species, locus-specific *F*_ST_ estimates involving the parental populations only (pop 1, pop 2, pop 8, pop 9) were calculated for the mtDNA locus (FSTAT 2.9.3.2) and AFLP loci (DFDIST). In order to assess population differentiation across the entire hybrid transect, *F*_ST_ for each locus was calculated (across all populations) in FSTAT 2.9 (mtDNA and isozyme loci) and DFDIST (AFLP loci).

We defined as clinal those loci that demonstrated a 20% or greater allele frequency change across all populations. Cline parameters were estimated according to a likelihood model (Szymura and Barton 1986) using the program Analyse 1.3 (Barton and Baird 1998). The program utilizes a sigmoid central curve and independent exponential decay curve tails to infer eight cline parameters of interest: center, width, splice points for the decay of each tail, the rate of decay of each tail, and maximum and minimum allele frequencies at each end of the transect (Brumfield et al. 2001). The fit of each locus to the inferred model is assessed via likelihood. Each locus was run through a 10,000 step Metropolis–Hastings fit optimization process 10 separate times to assure convergence of parameter estimates. Model equations and likelihood calculations are as described in Brumfield et al. (2001), although there was a typographical error in their Equation (3), corrected herein, in the Appendix.

Histograms of cline widths and centers estimated from the mtDNA, isozyme, and AFLP loci were compiled. Means and variances of the isozyme and AFLP parameter distributions were compared using ANOVA and F-tests, respectively. The sampling distribution of the cline parameters was explored using bootstrap model fits of three representative loci (mtDNA, TRI2, and AFLP 09_322); 10,000 additional Metropolis–Hastings iterations were run from which all model fits exhibiting likelihood values within two units of the maximized fit were sampled to create parameter confidence limits. The representative loci were chosen as exemplary models due to the characteristics of their model parameters: the mtDNA locus demonstrates a steep, sharp cline much like one that would result from intense selective pressure against hybrids. In fact, because the heterogametic sex in birds is female, if any locus were to reflect Haldane's rule, it would be the maternally inherited mitochondrial locus. The AFLP locus 09_322 was chosen to represent a “median model” because it exemplifies the observed median value for width among all loci and is very close to the median value for center. The TRI2 locus was selected as a “wide-shifted” exemplar. The sampling distribution of parameters for such different models might vary significantly from each other (and should cover much of the variation in the data), hence the choice of three, rather than a single exemplary model. The bootstrap resampling of the representative loci does not infer that one particular type of selection is responsible for any pattern; it instead provides a statistical method for assessing the variation associated with the characteristic cline shapes in order to test whether one type of model can account for all observed variation in the data.

Pearson's correlation coefficients were calculated to assess the relationship between cline width, cline center shift, and two types of locus-specific *F*_ST_ (calculated across parental populations and across all populations). Amplitude of cline center shift in either north or south direction from the mean was quantified using absolute value of [mean of all cline centers – locus cline center]. If loci that are highly differentiated between parental populations are more likely to exhibit reproductive incompatibilities between species, we should expect loci with large *F*_ST_ between parental populations to exhibit certain cline parameters (like narrow width). The second transect-wide estimate of *F*_ST_ across all populations should better reflect the pattern of transition through the hybrid transect; cline width and transect-wide *F*_ST_ should be measuring the same underlying structure at each locus across the hybrid zone.

To test the universality of cline parameters, pair-wise likelihood fits of each locus' empirical allele frequency data were fit to each of the cline models inferred for other loci. Each locus was used as both model and empirical data, resulting in a full matrix of log-likelihood fits. A full matrix of pair-wise log-likelihood fits for all loci was compiled (pair-wise fits calculated in *R*, http://www.r-project.org/). A principal co-ordinates analysis was performed on the asymmetrical pair-wise similarity matrix to assess clusters of similar loci; a custom R script which averages the off-diagonal components before executing the principal co-ordinates analysis was used (Gower 1966). The first two principal co-ordinate axes were plotted to identify any clustering in two-dimensional Euclidean distance space.

## Results

### Mitochondrial DNA

The mitochondrial locus is diagnostic in parental populations, with each individual bearing a fixed *maculatus* or *ocai* type pattern. This locus exhibits a prototypical steep clinal pattern, with cline width of 14.66 km and cline center at 841.54 km between populations 5 and 6, near the morphologically defined center of the hybrid zone (Braun 1983).

### Isozymes

Of the 39 isozyme loci, 28 are polymorphic and nine qualify as clinal, with none showing fixed differences. Mean cline width is broader than that for mtDNA at 197.29 km (range 40.33–450.57 km), whereas mean cline center is at 841.47 km (range 728.00–976.34 km).

### AFLP

Ten primer combinations render 377 polymorphic loci. Fifty-one of the AFLP loci qualify as clinal, with six showing fixed differences between at least two of the four parental populations. Mean cline width is 298.47 km (range 3.46–3303.57 km) and mean cline center is at 821.04 km (range 567.84–1145.39 km). Many of the individual birds demonstrate genetic intermediacy and backcross genotypes among the fixed markers, consistent with hybridization underlying the observed morphological variation rather than character displacement theory (Figure SA1).

### Analyses of cline position and shape

**Figure 3:**
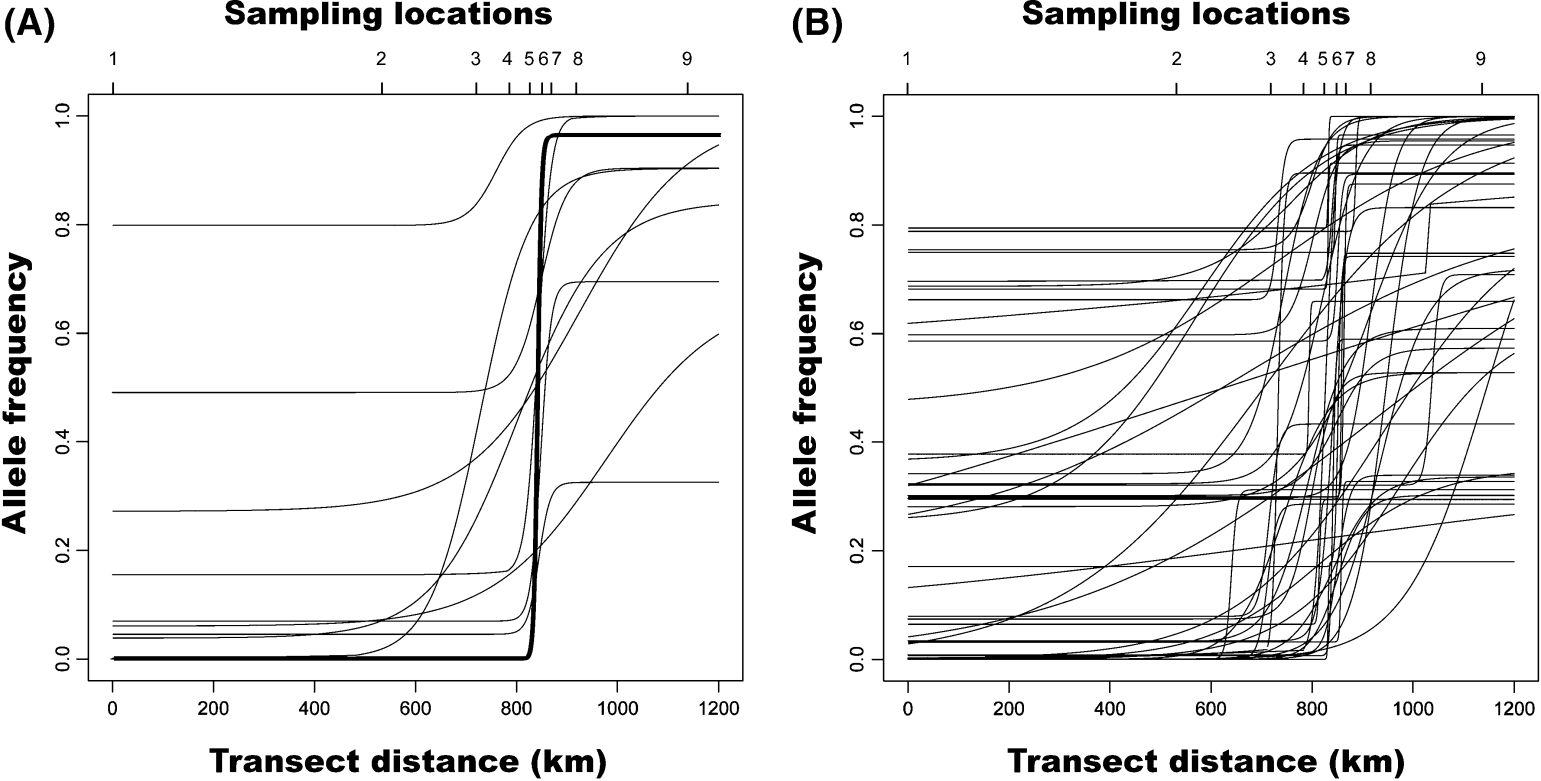
Cline models fit to empirical data for all 61 clinal loci using Analyse 1.3. Panel A shows the mtDNA (bold) and nine isozyme loci, whereas panel B shows the 51 AFLP loci. Sampling locations are noted on the secondary *X*-axis starting with population 1 at 0 km transect distance.

**Figure 4:**
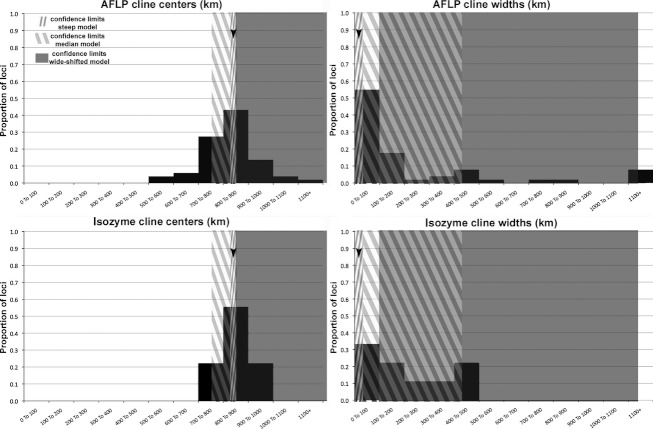
Distributions of cline centers and widths for AFLP (51) and isozyme (9) loci. Mitochondrial locus position is indicated by arrows. 95% confidence intervals for the bootstrap support on three empirical models (steep, median, and wide-shifted) are indicated by shading or cross-hatching.

Model fits to each of the loci reveal extensive variation in cline position and shape (Fig. 3). Histograms of the inferred parameters cline width and cline center for all 61 clinal loci (mtDNA locus + 9 isozyme loci + 51 AFLP loci) demonstrate this variation quantitatively (Fig. 4). Analysis of variance reveals no significant difference between isozyme and AFLP loci for either mean cline width or mean cline center. However, the variances of the cline width distributions differ significantly between isozyme and AFLP data due to the longer tail in the AFLP distribution consisting of loci corresponding to wide clines (*F*-test, *P* = 0.0008). The variances of the center distributions do not differ significantly. The pooled cline center distribution is normal (Shapiro–Wilk W *P* = 0.08, D'Agostino Kurtosis *P* = 0.08, D'Agostino Skewness *P* = 0.67).

Differentiation among parental populations (parental *F*_ST_) is not significantly correlated with either cline center shift or width. Cline center shift and cline width are significantly positively correlated; cline width and transect-wide locus-specific *F*_ST_ are significantly negatively correlated (Table 3). Wider clines tend to be more highly shifted from the hybrid zone center and tend to have lower cross-transect *F*_ST_ values; the lower levels of population differentiation and wider clines are reflecting the same underlying structure across the hybrid transect. The great majority of the measured loci do not exhibit fixed differences among species. Many clines that exhibit relatively low change in empirical allele frequency (Δp) also exhibit quite steep clines (Figure SA3). Although it is inherent that *F*_ST_ be correlated with Δp, it is not inherent that cline width should be correlated with Δp (and indeed it is not significantly correlated, Pearson's correlation coefficient= −0.16, *P* = 0.21). Because Δp and cline width are not confounded in this data set of mostly non-fixed loci, and the cline width parameter is independent of the inferred p_max_ and p_min_ parameters (Appendix), a system-wide correction for Δp is not warranted.

**Table 3:** Pearson correlation coefficients (lower diagonal) and associated p-values (upper diagonal) for pair-wise comparisons of cline center shift, width, and both parental and transect-wide *F*_ST_

	Center shift	Width	Parental *F*_ST_	Transect- wide *F*_ST_
Center shift	1	*0.010*	*0.600*	*0.528*
Width	0.329	1	*0.227*	*0.043*
Parental *F*_ST_	0.074	−0.170	1	*1.5 e-11*
Transect- wide *F*_ST_	−0.082	−0.260	0.775	1

Three exemplary models spanning the range of inferred parameters reveal that sampling error alone cannot explain the observed variation among loci in cline extent and shape. Confidence limits (95% CI) for the exemplary models for cline center (*c*) and width (*w*) are: 833.07 km < steep model *c* < 845.87 km, 0.60 km < steep model *w* < 34.56 km (4612 model fits within 2 log-likelihoods of the maximized fit); 755.47 km < median model *c* < 848.23 km, 6.92 km < median model *w* < 418.90 km (9147 model fits within 2 log-likelihoods of the maximized fit); 843.67 km < wide-shifted model *c* < 1261.03 km, 97.96 km < wide-shifted model *w* < 1135.43 km (6944 model fits within 2 log-likelihoods of the maximized fit). All sets of confidence limits are plotted in relation to all loci on Figure 4. Only 10% of all loci center parameter estimates (six loci) fall inside the steep model center support limits, 37% of all loci center parameter estimates (22 loci) fall inside the median model center support limits, whereas 42% of all loci center estimates (25 loci) fall inside the wide-shifted model support limits. Thirty-percent of all loci width estimates (18 loci) fall inside the steep model center support limits, 65% of all loci width estimates (39 loci) fall inside the median model width support limits, whereas only 43% of all loci width estimates (26 loci) fall within the wide-shifted model support limits.

**Figure 5:**
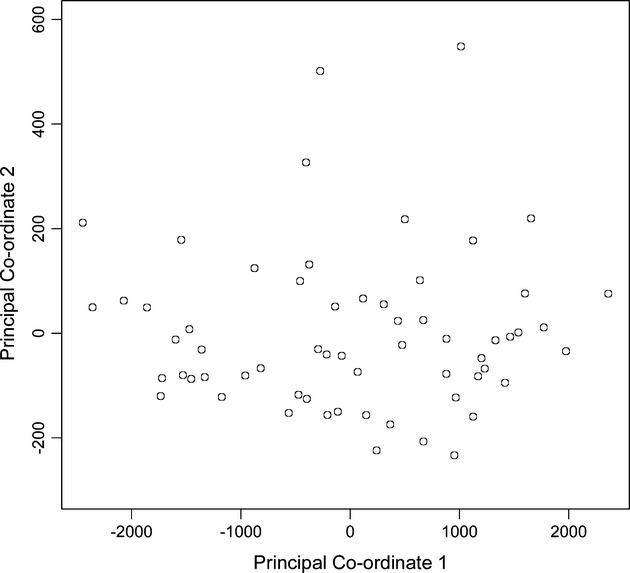
Principal co-ordinates in two dimensions performed on the asymmetrical log likelihood model fit matrix of the 61 different clinal loci.

The principal co-ordinates analysis of pair-wise model fits among all loci does not demonstrate any clearly defined groupings among the loci like one would expect if groups of loci were governed similarly by only a few sets of constraints. On the two-dimensional principal co-ordinates plot, PC1 accounts for 67.34% of the variance, whereas PC2 accounts for 8.62% (Fig. 5). In order to summarize empirical and model fit variation among loci, we plot graphs of each locus' empirical cline data (Figure SA2) as well as present model-inferred cline parameters for all loci (Table SA2).

## Discussion

Our multi-locus analysis demonstrates definitively that the morphological variation observed in this classic system is due to hybridization and not to release from character displacement or social mimcry between *P. maculatus* and *P. ocai*. If character displacement were driving the observed patterns, intermediate populations should be genetically like *maculatus* (Brown and Wilson 1956; Braun 1983). Instead, the populations in the middle of the Teziutlán gradient reveal extensive genetic admixture, as expected with hybridization (Figure SA1). Clarification of the hybrid status of these populations allows us to focus on the remarkable features of this system, which has been viewed as a classic case of the breakdown of reproductive isolation (Mayr 1963).

With a broad sampling of loci in hand, it is clear that significant variation exists among the clinal loci across the towhee hybrid gradient. The archetypal steep cline model cannot describe change in gene frequency across space for all loci. Even a median or wide-shifted model cannot fully encompass alone the magnitude of variance observed in these data. In addition, the heterogeneity observed lacks a discrete number of empirically evident groups, suggesting the underlying forces driving this variation do not have a strong cohesive cross-locus signal (Fig. 5). We demonstrate not only significant variation in cline model parameters and corresponding clines shapes among loci across the genome, but also a negative correlation between wide clines and locus-specific differentiation (transect-wide *F*_ST_) across the gradient. Similar lack of co-incidence among cline centers has been observed in the North American hybrid zone between glaucous-winged gulls and western gulls; however, the extent of variation in cline width we observe herein is not matched in the gull hybrid zone (Gay et al. 2008).

While the mitochondrial locus falls near the mean and mode in center and width parameters, we observe substantial variance in both the isozyme and AFLP loci (Figs 3 and 4). Similar nuclear variation from the mitochondrial signal has also been demonstrated in a patchy butterfly hybrid zone (Gompert et al. 2008). Although many towhee loci exhibit cline centers shifted from the mean, there is no bias in direction of shift as one would expect in a mobile rather than stable hybrid zone (Fig. 4). The lack of correlation between parental *F*_ST_ and cline width suggests that the more highly differentiated loci are not solely representing highly constrained loci (likely to exhibit incompatibilities). This pattern may result from the observed loci existing in equilibrium state due to the age and stability of the hybrid contact; over time, breakdown of linkage disequilibrium may reduce the effect of incompatibilities. In addition, some loci highly differentiated among parentals may reflect patterns created by demographic-oriented processes across the hybrid transect, like isolation by distance.

The loci that exhibit the classic sharp cline morphology are likely constrained against gene flow due to the balance between dispersal and selection. Much like the mitochondrial locus, plumage characters in the towhee Teziutlán gradient exhibit steep transitions and are likely under strong selective pressure (Braun 1983). In the case of the mitochondrial locus, Haldane's rule could contribute to this strong transition, as avian females are the heterogametic sex (Haldane 1922). However, there is clear cross-genomic variation in selective constraint and locus-specific flexibility in the permeability of the interspecies membrane (Fig. 4). Both exogenous and endogenous selective factors could be important driving factors for these loci; coupling between endogenous and exogenous barriers to gene flow even in the presence of genetic-environment association has been demonstrated theoretically (Bierne et al. 2011). Sex-linked loci are often focal in hybrid zone studies as they are likely to be under greater selective pressure (Payseur et al. 2004; Macholán et al. 2007; Carling and Brumfield 2008). Although our analyses do not elucidate a tightly associated group of loci with narrow, steep clines, the sex-linkage status of our anonymous loci warrants further exploration.

The lack of natural groupings of loci demonstrated using the principal co-ordinates plot suggests that while we have sampled a cross-section of among-locus variation, loci do not naturally fall into easily defined categories based on similarities in cline extent and shape. Differentiation and gene flow across the genome is not uniform or easily binned into a small number of categories. Even though the hybrid zone itself appears geographically stable, some alleles are free to introgress across species, whereas others are highly constrained. This lack of uniformity in cline structures suggests that among-locus variation in introgression is high but not categorical, which is relevant to the “islands of differentiation” concept (Harr 2006; Nosil et al. 2008; Feder and Nosil 2010; Nadeau et al. 2012). If loci important to maintaining reproductive isolation between these two species were tightly linked and clustered into a small number of genomic islands, we would expect the observed variation to exhibit at least some natural groupings. The lack of natural groupings suggests that this system does not exhibit a strong signal for a small number of islands of differentiation. Although evidence of similar levels of heterogeneity in differential introgression is prevalent in the hybrid literature (Macholán et al. 2007; Carling and Brumfield 2008; Teeter et al. 2008; Yuri et al. 2009; Carling and Zuckerberg 2011; Dufkova et al. 2011), searching for clusters of loci (or lack thereof) in the absence of a genome or linkage map is a unique aspect of this analysis.

Given the likely age of the secondary contact between *P. maculatus* and *P. ocai*, one might hypothesize that the less constrained alleles would sweep across the hybrid zone, erasing some portion of the original differentiation between these taxa. Although the movement would occur very quickly for advantageous alleles, gene flow could eventually bring across neutral alleles as well (Macholán et al. 2007). So why have not all the unconstrained alleles swept across the zone and further increased homogeneity between the two species? One possibility is that local variation in habitat and the island-like distribution of habitat patches along the hybrid gradient contribute to local differentiation. Drift and differential selection within semi-isolated populations complicates long distance gene flow across the species. Differentiation in hybrid populations is often observed in mosaic hybrid zones, but is less commonly emphasized in classic hybrid transects where greater landscape continuity may facilitate gene flow (Vines et al. 2003; Mallet et al. 2007; Yuri et al. 2009; Gompert et al. 2010b). Barriers to habitat continuity show the potential to stifle introgression in a manakin hybrid zone in Panama (Brumfield 1999; Stein and Uy 2006). Habitat patchiness related to elevation and availability of scrubby pine/oak forest could influence local population isolation and corridors of gene flow in the Mexican towhee hybrid zone.

Clines within the *P. maculatus* – *P. ocai* Teziutlán hybrid gradient reveal cross-genomic heterogeneity indicating significant locus-to-locus variation in the porosity of the species membrane. The presence of loci in many phases of constrained and unconstrained gene flow suggests that even within an old, stable area of contact, differential introgression is an ongoing fluid and dynamic process. This potential for evolutionarily significant differential introgression across species boundaries bears importantly on our interpretation of species concepts. Incomplete reproductive isolation and divergence with gene flow may not just be relevant in the context of incipient speciation, but also for older lineages in and out of contact cyclically (Broyles 2002; Gay et al. 2008). Although regular instances of introgressive hybridization may add a level of variance to the classic and beautiful model of bifurcating lineages, it also reaffirms our need for extensive multi-locus datasets for inference in both population and phylogenetic fields.

## Appendix

Here we present the equations for the tension zone-based cline model that can accommodate spatial shifts in cline location as well as changes in changes in cline shape. Model equations and likelihood calculations are from Brumfield et al. (2001), but are re-presented here to correct a typographical error in Equation 3 of that paper. Along a sampled transect, the estimated frequency of the modeled allele, *p*_*est*_*,* is described piecewise by a three-part function (Szymura and Barton 1986, 1991):

(A1)

where ,,  , , *t*_*N*_ = *β*_*N*_*decay*_*N*_, and *t*_*S*_ = *β*_*S*_*decay*_*S*_. Note that the position *x* along the transect (measured in km from the northernmost population) gets rescaled for use in equation 1 according to , where the cline width,  and the cline center is *c*. We are interested in a total of eight cline parameters. In addition to *w* and *c*, we seek *θ*_*N*_ and *θ*_*S*_ (the northern and southern tail splice parameters, respectively), β_N_ and β_S_ (shape parameters governing the decay of the northern and southern tails, respectively), plus *p*_*min*_ and *p*_*max*_ (the lowest and highest modeled allele frequencies, respectively). To obtain maximum likelihood estimates of the parameters, we rescale the allele frequencies according to

(A2)

and calculate

(A3)

where *n* is the number of alleles sampled in the population and *p*_*obs*_ is the observed frequency of the modeled allele at position *X*. The total model support is then the sum of population-level log-likelihoods.

## Supporting Information

**Figure SA1.** Non-metric multidimensional scaling analysis in two dimensions derived from a Jaccard similarity matrix based on the binary AFLP scores at 377 polymorphic loci for all individuals. Individuals are color-coded by sampling population.

**Figure SA2.** Empirical data for all 61 clinal loci displayed in vertically adjacent plots. Populations extend across the *X*-axis; each pane's *Y*-axis represents allele frequency.

**Figure SA3.** Exemplary cline fits where change in allele frequency over the transect is relatively small, but cline width is narrow.

**Table SA1.** Tissue and voucher specimen numbers listed by population. Tissue samples were deposited at the US National Museum of Natural History and vouchers were deposited at Louisiana State University Museum of Natural Science.

**Table SA2.** Inferred cline parameters for all loci and locus-specific F_ST_.
