# Supplementary material for: Genomic variation in cline shape across a hybrid zone
Source: Ecol Evol. 2012 Oct 1;2(11):2737–48. doi: 10.1002/ece3.375 (PMC3501626; doi:10.1002/ece3.375)
Supplement: Supplementary file 1 [file ece30002-2737-SD1.pdf]

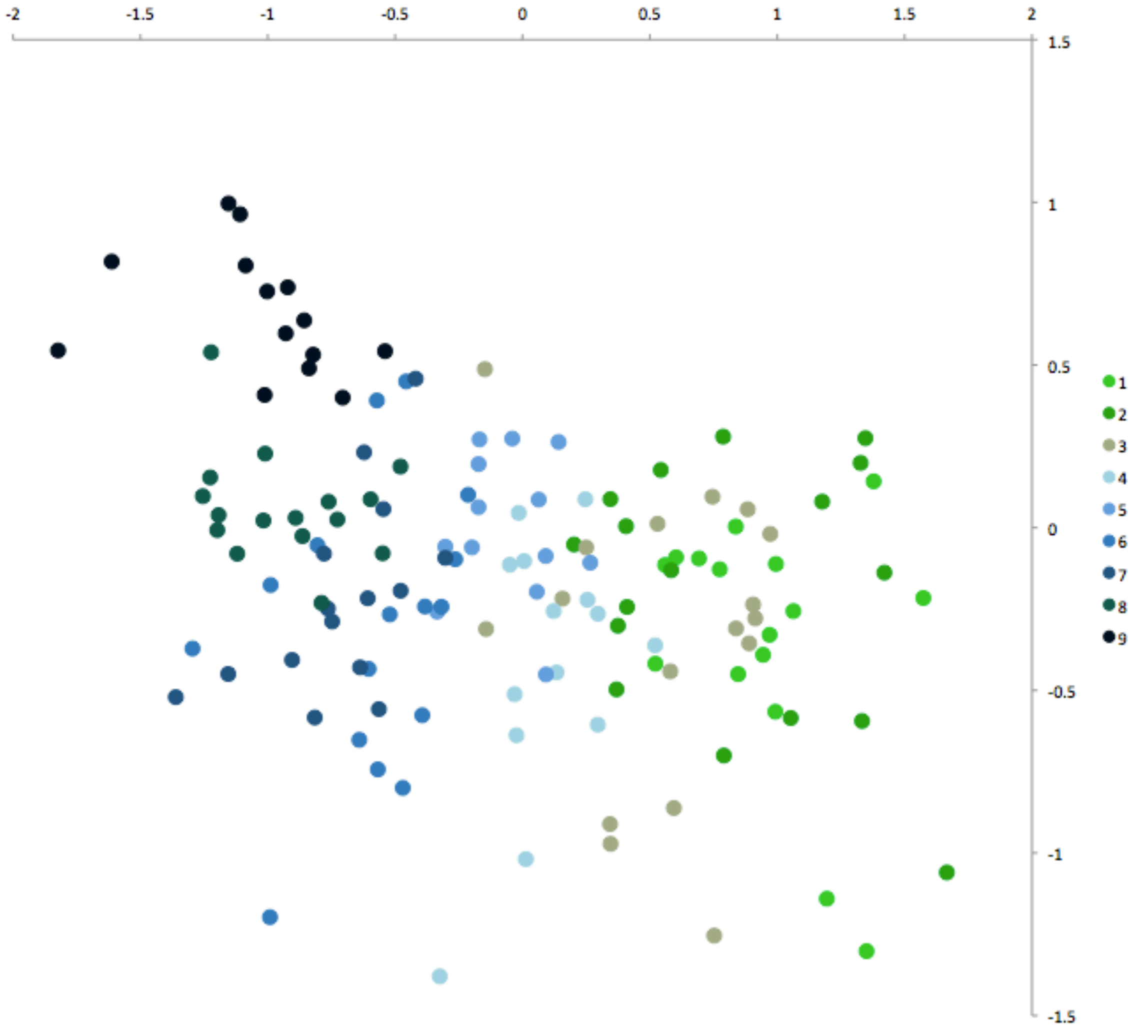

Appendix Figure 1. Non-metric multidimensional scaling analysis in two dimensions derived from a Jaccard similarity matrix based on the binary AFLP scores at 377 polymorphic loci for all individuals. Individuals are color-coded by sampling population.
