# Supplementary material for: Genomic variation in cline shape across a hybrid zone
Source: Ecol Evol. 2012 Oct 1;2(11):2737–48. doi: 10.1002/ece3.375 (PMC3501626; doi:10.1002/ece3.375)
Supplement: Supplementary file 2 [file ece30002-2737-SD2.pdf]

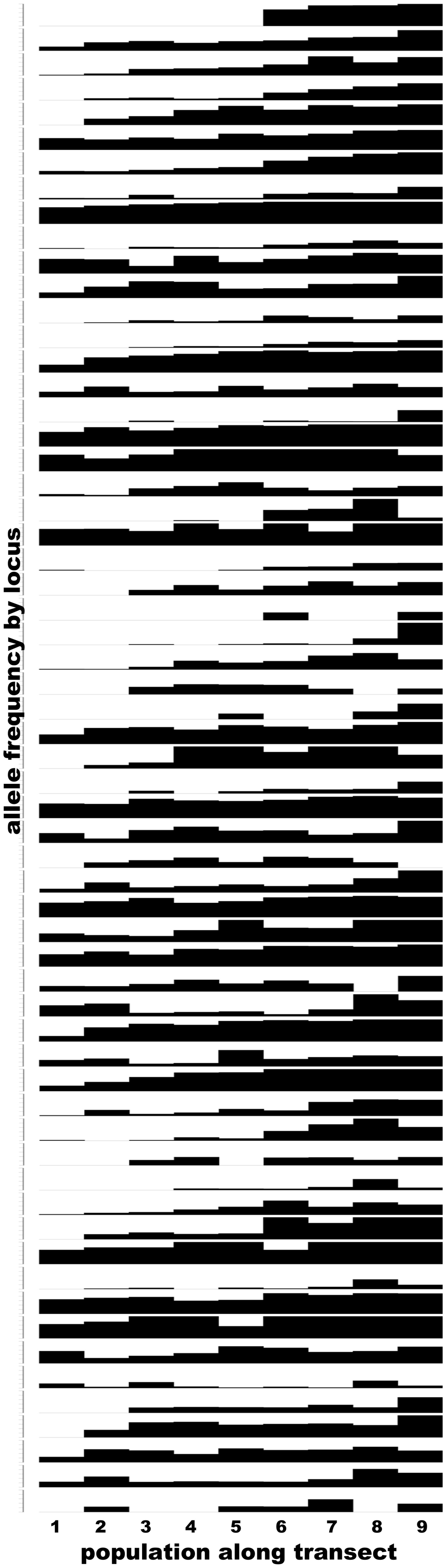

Appendix Figure 2. Empirical data for all 61 clinal loci displayed in vertically adjacent plots. Populations extend across the X-axis; each pane's Y-axis represents allele frequency.
