# Supplementary material for: Genomic variation in cline shape across a hybrid zone
Source: Ecol Evol. 2012 Oct 1;2(11):2737–48. doi: 10.1002/ece3.375 (PMC3501626; doi:10.1002/ece3.375)
Supplement: Supplementary file 3 [file ece30002-2737-SD3.pdf]

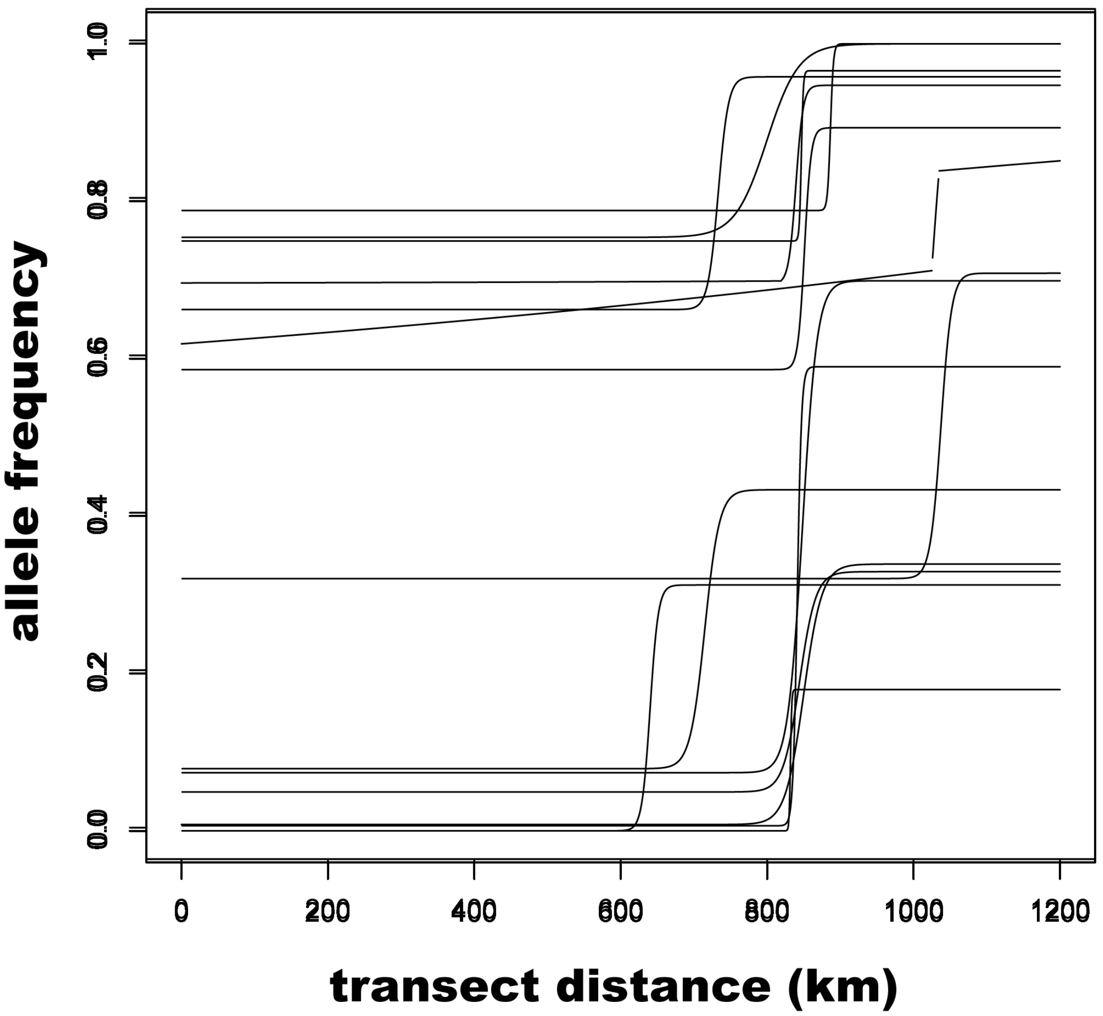

Appendix Figure 3. Exemplary cline fits where change in allele frequency over the transect is relatively small, but cline width is narrow.

Appendix Table 1. Tissue and voucher specimen numbers listed by population. Tissue samples were deposited at the US National Museum of Natural History and vouchers were deposited at Louisiana State University Museum of Natural Science.

USNM Tissue No.	Voucher No.	Population
B00625	LSUMZ101112	Coahuila
B00626	LSUMZ101109	Coahuila
B00627	LSUMZ101110	Coahuila
B00628	LSUMZ101113	Coahuila
B00629	LSUMZ101114	Coahuila
B00630	LSUMZ101119	Coahuila
B00631	LSUMZ101121	Coahuila
B00632	LSUMZ101115	Coahuila
B00633	LSUMZ101120	Coahuila
