# Supplementary material for: Genomic variation in cline shape across a hybrid zone
Source: Ecol Evol. 2012 Oct 1;2(11):2737–48. doi: 10.1002/ece3.375 (PMC3501626; doi:10.1002/ece3.375)
Supplement: Supplementary file 4 [file ece30002-2737-SD4.pdf]

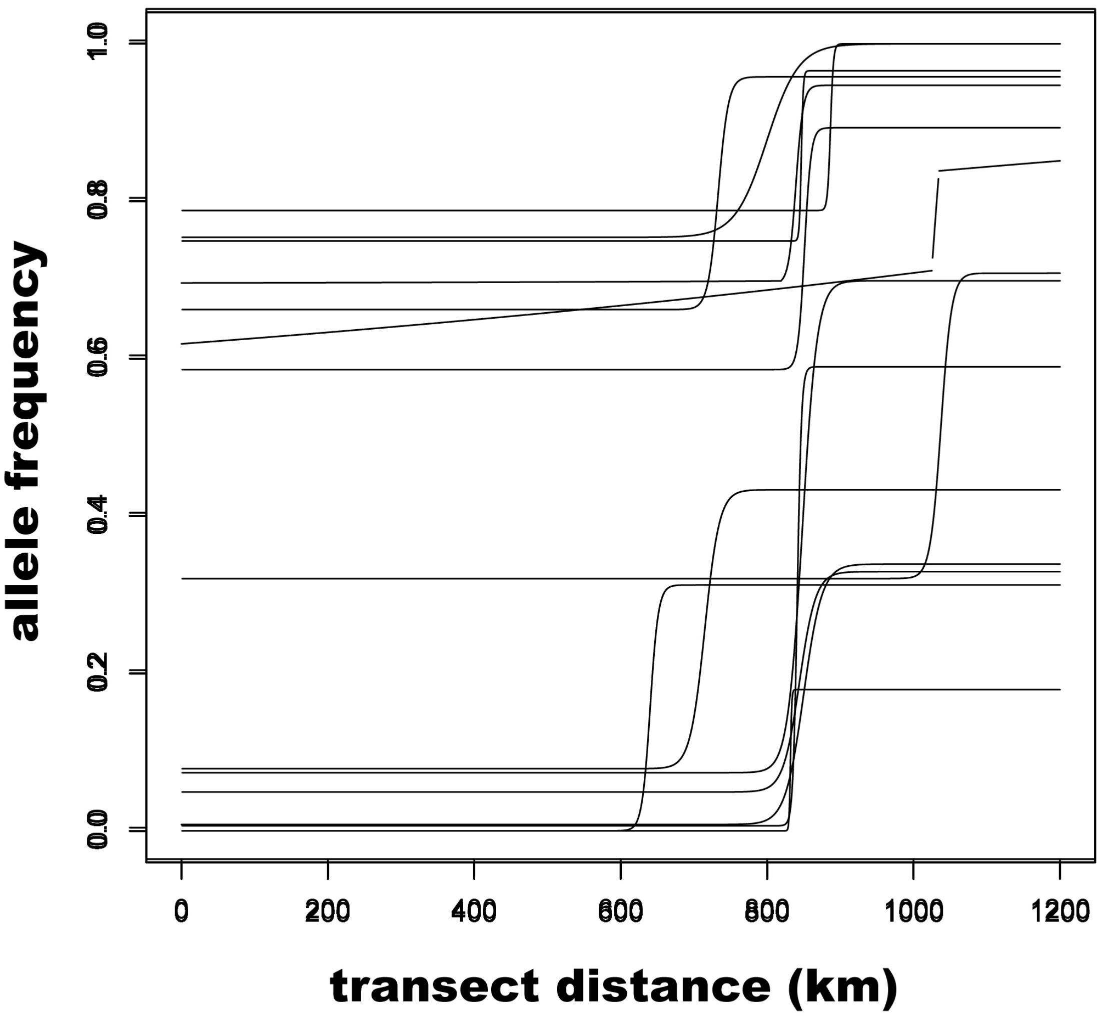

Appendix Figure 3. Exemplary cline fits where change in allele frequency over the transect is relatively small, but cline width is narrow.

Appendix Table 1. Tissue and voucher specimen numbers listed by population. Tissue samples were deposited at the US National Museum of Natural History and vouchers were deposited at Louisiana State University Museum of Natural Science.

USNM Tissue No.	Voucher No.	Population
B00625	LSUMZ101112	Coahuila
B00626	LSUMZ101109	Coahuila
B00627	LSUMZ101110	Coahuila
B00628	LSUMZ101113	Coahuila
B00629	LSUMZ101114	Coahuila
B00630	LSUMZ101119	Coahuila
B00631	LSUMZ101121	Coahuila
B00632	LSUMZ101115	Coahuila
B00633	LSUMZ101120	Coahuila

USNM Tissue No.	Voucher No.	Population
B00634	LSUMZ101122	Coahuila
B00635	LSUMZ101111	Coahuila
B00636	LSUMZ101116	Coahuila
B00637	LSUMZ101117	Coahuila
B00638	LSUMZ101123	Coahuila
B00639	LSUMZ101118	Coahuila
B00640	LSUMZ101146	Coahuila
B00641	LSUMZ101124	Queretaro
B00642	LSUMZ101128	Queretaro
B00643	LSUMZ101076	Queretaro
B00644	LSUMZ101127	Queretaro
B00645	LSUMZ101125	Queretaro
B00646	LSUMZ101126	Queretaro
B00647	LSUMZ101147	Queretaro
B00648	LSUMZ101132	Queretaro
B00649	LSUMZ101129	Queretaro
B00650	LSUMZ101131	Queretaro
B00651	LSUMZ101133	Queretaro
B00652	LSUMZ101130	Queretaro
B00653	LSUMZ101134	Queretaro
B00654	LSUMZ101137	Queretaro
B00655	LSUMZ101136	Queretaro
B00656	LSUMZ101135	Queretaro
B00657	LSUMZ101138	Queretaro
B00067	LSUMZ91346	Tlaxco
B00068	LSUMZ91347	Tlaxco
B00069	LSUMZ91348	Tlaxco
B00070	LSUMZ91349	Tlaxco
B00071	LSUMZ91350	Tlaxco
B00072	LSUMZ91351	Tlaxco
B00658	LSUMZ101077	Tlaxco
B00659	LSUMZ101081	Tlaxco
B00660	LSUMZ101082	Tlaxco
B00661	LSUMZ101083	Tlaxco
B00662	LSUMZ101079	Tlaxco
B00663	LSUMZ101080	Tlaxco
B00664	LSUMZ101084	Tlaxco
B00665	LSUMZ101078	Tlaxco
B00666	LSUMZ101148	Tlaxco
B00667	LSUMZ101086	Tlaxco
B00668	LSUMZ101085	Tlaxco
B00001	LSUMZ91332	E. Huichautla
B00002	LSUMZ91333	E. Huichautla
B00006	LSUMZ91334	E. Huichautla
B00007	LSUMZ91335	E. Huichautla
B00008	LSUMZ91336	E. Huichautla

USNM Tissue No.	Voucher No.	Population
B00009	LSUMZ91337	E. Huichautla
B00010	LSUMZ91338	E. Huichautla
B00011	LSUMZ91339	E. Huichautla
B00032	LSUMZ91340	E. Huichautla
B00033	LSUMZ91341	E. Huichautla
B00034	LSUMZ91342	E. Huichautla
B00035	LSUMZ91343	E. Huichautla
B00036	LSUMZ91344	E. Huichautla
B00037	LSUMZ91345	E. Huichautla
B00012	LSUMZ91325	Teziutlán
B00019	LSUMZ91326	Teziutlán
B00020	LSUMZ91327	Teziutlán
B00021	LSUMZ91328	Teziutlán
B00038	LSUMZ91317	Teziutlán
B00039	LSUMZ91318	Teziutlán
B00040	LSUMZ91319	Teziutlán
B00041	LSUMZ91320	Teziutlán
B00042	LSUMZ91321	Teziutlán
B00046	LSUMZ91329	Teziutlán
B00047	LSUMZ91330	Teziutlán
B00049	LSUMZ91331	Teziutlán
B00050	LSUMZ91322	Teziutlán
B00051	LSUMZ91323	Teziutlán
B00052	LSUMZ91324	Teziutlán
B00004	LSUMZ91316	R. Palenquillo
B00014	LSUMZ91304	R. Palenquillo
B00015	LSUMZ91305	R. Palenquillo
B00017	LSUMZ91306	R. Palenquillo
B00018	LSUMZ91307	R. Palenquillo
B00059	LSUMZ91304	R. Palenquillo
B00060	LSUMZ91305	R. Palenquillo
B00061	LSUMZ91310	R. Palenquillo
B00062	LSUMZ91311	R. Palenquillo
B00063	LSUMZ91312	R. Palenquillo
B00064	LSUMZ91313	R. Palenquillo
B00065	LSUMZ91314	R. Palenquillo
B00066	LSUMZ91315	R. Palenquillo
B00669	LSUMZ101058	R. Palenquillo
B00670	LSUMZ101075	R. Palenquillo
B00671	LSUMZ101074	R. Palenquillo
B00003	LSUMZ91289	Cofre Perote
B00005	LSUMZ91290	Cofre Perote
B00024	LSUMZ91291	Cofre Perote
B00025	LSUMZ91292	Cofre Perote
B00026	LSUMZ91293	Cofre Perote
B00027	LSUMZ91294	Cofre Perote

USNM Tissue No.	Voucher No.	Population
B00028	LSUMZ91295	Cofre Perote
B00029	LSUMZ91296	Cofre Perote
B00030	LSUMZ91297	Cofre Perote
B00053	LSUMZ91298	Cofre Perote
B00054	LSUMZ91299	Cofre Perote
B00055	LSUMZ91300	Cofre Perote
B00056	LSUMZ91301	Cofre Perote
B00057	LSUMZ91302	Cofre Perote
B00058	LSUMZ91303	Cofre Perote
B00676	LSUMZ101043	Orizaba
B00679	LSUMZ101044	Orizaba
B00683	LSUMZ101045	Orizaba
B00684	LSUMZ101046	Orizaba
B00688	LSUMZ101051	Orizaba
B00689	LSUMZ101048	Orizaba
B00690	LSUMZ101049	Orizaba
B00691	LSUMZ101050	Orizaba
B00692	LSUMZ101047	Orizaba
B00695	LSUMZ101055	Orizaba
B00696	LSUMZ101052	Orizaba
B00697	LSUMZ101056	Orizaba
B00698	LSUMZ101054	Orizaba
B00699	LSUMZ101073	Orizaba
B00700	LSUMZ101053	Orizaba
B00701	LSUMZ101057	Orizaba
B00703	LSUMZ101060	Oaxaca
B00704	LSUMZ101067	Oaxaca
B00705	LSUMZ101063	Oaxaca
B00706	LSUMZ101065	Oaxaca
B00707	LSUMZ101064	Oaxaca
B00709	LSUMZ101066	Oaxaca
B00710	LSUMZ101061	Oaxaca
B00711	LSUMZ101059	Oaxaca
B00712	LSUMZ101068	Oaxaca
B00714	LSUMZ101071	Oaxaca
B00716	LSUMZ101070	Oaxaca
B00717	LSUMZ101069	Oaxaca
B00718	LSUMZ101480	Oaxaca
B00723	LSUMZ101072	Oaxaca
