# Supplementary material for: Genomic variation in cline shape across a hybrid zone
Source: Ecol Evol. 2012 Oct 1;2(11):2737–48. doi: 10.1002/ece3.375 (PMC3501626; doi:10.1002/ece3.375)
Supplement: Supplementary file 5 [file ece30002-2737-SD5.pdf]

Appendix Table 2.
Inferred cline parameters for all loci and locus-specific F_{ST}

locus	Type	center	width	theta N	theta S	BN	BS	pmin	pmax	transect-wide F_{ST}
mtDNA	mtDNA RFLP	841.54	14.66	0.0145	0.7567	25014685735	117314165095	0.0001	0.9642	0.859
DIP1	isozyme	923.00	436.11	0.0683	0.1255	2629207686972	1471177871213	0.2719	0.9999	0.122
GOT1	isozyme	802.45	337.90	0.4025	0.1805	997679893923	3070931114324	0.0384	0.8434	0.283
EDH	isozyme	848.29	40.33	0.7872	9305269248	215854781821	7	0.0698	0.6947	0.379
TF	isozyme	728.00	200.74	0.1175	0.1948	85	568090617618	0.0001	0.9035	0.424
EST2	isozyme	848.36	102.49	0.3629	0.8888	88	500401715077	0.4908	0.9041	0.119
GPI	isozyme	842.77	51.05	0.2269	0.3218	40	36	0.1551	0.9999	0.332
TRI2	isozyme	976.34	450.57	0.9697	0.5055	4214766170223	3826153990072	0.0606	0.6721	0.155
6PGD	isozyme	759.81	112.04	0.2564	0.6586	181969208310	927608439613	0.7985	0.9999	0.021
DIP2	isozyme	844.21	44.40	0.5625	0.7993	338662616126	374616181516	0.0453	0.3252	0.094
01_114	AFLP	851.07	17.83	0.2084	0.8584	19361033857	65119090017	0.5861	0.8933	0.293
01_141	AFLP	917.00	283.80	0.1611	0.9907	256	2110742974933	0.2809	0.9999	0.147
01_148	AFLP	816.44	449.71	0.0144	0.7560	2160840609	980268095	0.0001	0.3536	0.101
01_203	AFLP	842.79	169.82	0.3931	0.8680	298204932220	752872214961	0.0001	0.3357	0.124
01_220	AFLP	582.39	472.52	0.9702	0.1638	4156905602604	1624572537086	0.3641	0.9999	0.277
01_409	AFLP	666.91	1102.91	0.9770	0.9999	6052193617971	21	0.2164	0.8339	0.040
02_215	AFLP	1145.39	322.91	1.0000	0.0049	343	3975	0.0001	0.9982	0.301
02_333	AFLP	799.60	86.96	0.4281	0.4179	312944186829	286059191063	0.7541	0.9999	0.134
02_342	AFLP	733.17	27.81	0.3149	0.9636	107965818220	15167009022	0.6623	0.9580	0.221
03_140	AFLP	714.84	40.00	0.9928	0.9974	3606	1211	0.0790	0.4334	0.089
03_402	AFLP	841.16	11.52	0.9617	0.7706	48538338639	90299182601	0.0065	0.5897	0.649
03_407	AFLP	886.53	8.00	0.4778	0.4957	34119456739	19377420290	0.7881	0.9999	0.177
04_125	AFLP	849.91	50.06	0.9398	0.9738	84	480	0.0081	0.3390	0.166
04_130	AFLP	743.66	189.04	0.3543	0.6820	1532682836951	1888792059802	0.0001	0.5282	0.276
04_218	AFLP	831.83	3.46	0.0043	0.1881	647462468	31618008613	0.0001	0.1795	0.321

locus	Type	center	width	theta N	theta S	BN	BS	pmin	pmax	transect-wide F_{ST}
04_428	AFLP	946.40	130.77	0.8888	0.3629	638482121986	113	0.0001	0.9999	0.551
05_120	AFLP	790.75	149.75	0.2173	0.7940	1098557461678	642515851281	0.0312	0.6093	0.264
05_187	AFLP	640.17	22.37	0.6656	0.1259	75479880620	139826931125	0.0001	0.3124	0.226
05_190	AFLP	934.30	188.84	4619326976	2573597440	109	62	0.0001	0.7186	0.458
05_216	AFLP	730.53	809.00	0.6320	0.6660	4667265447321	5180115450923	0.4648	0.9999	0.104
05_225	AFLP	732.34	22.50	0.0026	0.3166	442	84055321352	0.0001	0.8956	0.546
05_240	AFLP	973.32	439.78	0.1502	0.3162	753744322942	3745930469394	0.0001	0.6349	0.191
05_258	AFLP	846.70	5.22	0.3801	0.7685	49917466010	23985803914	0.7494	0.9657	0.070
09_165	AFLP	1026.90	40.06	0.0000	0.0000	27	39	0.4796	0.9999	0.141
09_215	AFLP	917.00	1217.73	1.0000	1.0000	1218	1218	0.0001	0.8751	0.167
09_217	AFLP	917.31	99.39	0.9468	0.9212	99	99	0.2964	0.9999	0.247
09_232	AFLP	839.29	19.78	0.0002	0.4609	2333	41245963	0.6950	0.9472	0.066
09_287	AFLP	819.92	160.98	0.7992	0.8365	1444386498490	1079119584783	0.3232	0.9999	0.297
09_322	AFLP	802.08	92.95	0.8371	0.4432	918523525571	374496772682	0.5977	0.9550	0.171
10_192	AFLP	1037.47	29.26	0.9357	0.0870	175867	1098	0.3206	0.7086	0.219
10_194	AFLP	863.04	7.95	0.4494	0.1182	1290623850	67133132194	0.2990	0.8753	0.469
10_216	AFLP	567.84	497.88	0.2305	0.2612	3461912019282	1112863523894	0.2532	0.9999	0.294
10_288	AFLP	848.61	144.91	0.9997	0.9891	324	160401	0.3017	0.5729	0.146
10_332	AFLP	743.05	138.43	0.2958	0.6210	364450853661	591988341230	0.3420	0.9999	0.396
10_395	AFLP	851.55	12.18	0.3856	0.6984	54882868803	93274107327	0.1707	0.7420	0.256
11_140	AFLP	840.48	52.59	0.1078	0.4331	138382876630	426085388837	0.0340	0.8320	0.591
11_177	AFLP	711.86	37.38	0.8118	0.4175	134376572510	102622223582	0.0001	0.2857	0.127
11_216	AFLP	850.95	105.88	0.0055	0.9851	916037054300	677177499743	0.0001	0.3019	0.245
11_347	AFLP	798.67	90.14	0.0534	0.8992	54881956778	533210250414	0.0741	0.5279	0.152
12_109	AFLP	826.34	43.08	0.0027	0.5789	509	41	0.0001	0.9999	0.501
12_196	AFLP	760.81	332.31	0.3152	0.0527	2133099620383	2655234030043	0.6871	0.9999	0.177
12_207	AFLP	857.84	6.64	0.3748	0.7631	4911262285	48105947299	0.0320	0.3277	0.146
12_277	AFLP	831.77	3.71	0.6796	0.3690	24469771899	2651050797	0.6822	0.9137	0.134
13_137	AFLP	832.10	3.69	0.4217	0.7657	8048501307	6139812471	0.7943	0.9999	0.292
13_159	AFLP	794.09	5.23	0.1118	0.1921	4054573917	43662187994	0.3779	0.6591	0.127

locus	Type	center	width	theta N	theta S	BN	BS	pmin	pmax	transect-wide F_{ST}
13_194	AFLP	768.74	2382.19	0.0853	1.0000	3466152673965	54	0.0688	0.3624	0.076
13_226	AFLP	942.93	580.00	0.3654	0.2041	5272962061005	4647989098076	0.0001	0.8420	0.234
13_229	AFLP	704.25	796.18	0.1880	0.3205	796	796	0.0001	0.9999	0.249
13_243	AFLP	627.87	3303.57	1.0000	0.9999	3304	3304	0.0014	0.9999	0.099
13_264	AFLP	858.30	5.41	0.5714	0.6347	23342906074	53933760037	0.2950	0.7478	0.140
13_295	AFLP	814.66	7.94	0.3207	0.5834	26182761727	57761518464	0.0644	0.2943	0.270
